# Identification of a Thermostable Levansucrase from *Pseudomonas orientalis* That Allows Unique Product Specificity at Different Temperatures

**DOI:** 10.3390/polym15061435

**Published:** 2023-03-14

**Authors:** Cuie Guang, Xiaoqi Zhang, Dawei Ni, Wenli Zhang, Wei Xu, Wanmeng Mu

**Affiliations:** 1 State Key Laboratory of Food Science and Technology, Jiangnan University, Wuxi 214122, China; 2 International Joint Laboratory on Food Safety, Jiangnan University, Wuxi 214122, China

**Keywords:** levansucrase, thermostability, levan, product specificity, application

## Abstract

The biological production of levan by levansucrase (LS, EC 2.4.1.10) has aroused great interest in the past few years. Previously, we identified a thermostable levansucrase from *Celerinatantimonas diazotrophica* (Cedi-LS). A novel thermostable LS from *Pseudomonas orientalis* (Psor-LS) was successfully screened using the Cedi-LS template. The Psor-LS showed maximum activity at 65 °C, much higher than the other LSs. However, these two thermostable LSs showed significantly different product specificity. When the temperature was decreased from 65 to 35 °C, Cedi-LS tended to produce high-molecular-weight (HMW) levan. By contrast, Psor-LS prefers to generate fructooligosaccharides (FOSs, DP ≤ 16) rather than HMW levan under the same conditions. Notably, at 65 °C, Psor-LS would produce HMW levan with an average *M*_w_ of 1.4 × 10^6^ Da, indicating that a high temperature might favor the accumulation of HMW levan. In summary, this study allows a thermostable LS suitable for HMW levan and levan-type FOSs production simultaneously.

## 1. Introduction

Levansucrase (LS, EC 2.4.1.10), inulosucrase (IS, EC 2.1.4.9), and β-fructofuranosidase (Ffase, EC 3.2.1.26) are three fructansucrases (FSs) that could use sucrose as the substrate to produce fructooligosaccharides (FOSs) and fructans (homopolymers of fructose) [1]. LS and IS belong to the glycoside hydrolase 68 (GH 68) family of enzymes, while Ffase may be categorized as GH68 or GH32 family enzymes. These enzymes can all hydrolyze sucrose and subsequently synthesize fructan, which are defined as hydrolysis reaction (H) and transfructosylation (T), respectively [2]. Both reactions start from sucrose splitting into glucose and a fructosyl moiety. The “T” reaction occurs when the fructosyl moiety is transferred to an acceptor such as the sucrose or the elongating fructan chain. The “H” reaction will happen when the acceptor is water, releasing glucose and fructose [3].

LS, IS, and Ffase have distinguished product specificities. Ffase exclusively synthesizes FOS as its main product, whereas LS and IS could produce FOS and fructan. In addition, the product generated by IS, inulin, primarily consists of β-(2,1) linkages on the polymer backbone, while the fructan generated by LS, levan, harbors β-(2,6) linkages on the main chain [4]. Meanwhile, the identified LSs from more than 40 kinds of microorganisms all alternatively produce β-(2,6) type FOSs, β-(2,6)-type low-molecular-weight (LMW, FOS < *M*_W_ < 5 × 10^4^ Da) levan, and β-(2,6)-type high-molecular-weight (HMW, *M*_W_ > 5 × 10^4^ Da) levan in the reaction mixture. For instance, the LSs from *Erwinia amylovora* [5] and *Zymomonas* species [6] produced FOSs with a degree of polymerization (DP) below 10 as the main product. However, they only produced a small amount of HMW or LMW levan. The LS from *Bacillus methylotrophicus* SK 21.002 is the only one that produces LMW levan with an *M*_W_ of 4–5 × 10^3^ Da [7]. By contrast, the LSs from *Acetobacter nitrogenifigens* RG1 [8] and *Lactobacillus reuteri* LTH5448 [9] could synthesize HMW levan as the main product, with an *M*_W_ of 7.1 × 10^6^ and 3.9 × 10^7^ Da, respectively. In particular, the LS from *Clostridium acetobutylicum* could exclusively synthesize levan rather than FOSs in the reaction [10].

The properties of levan were varied with *M*_W_. For instance, the LMW levan was reported to have a potential role in peptic ulcer curing [11] and carcinogenesis initiation stage inhibitory [12], while HMW levan could act as an antivirus agent [13] and pancreatic anticancer agent [14]. Since the practical application of levan dramatically depends on its *M*_W_, many attempts have been made to explore the potential reason for the product specificity of LSs. Enzyme concentration was regarded as a critical factor. For instance, the LS from *Bacillus subtilis* produced LMW levan (*M*_W_ = 7.2 × 10^3^ Da) at a high enzyme concentration (10 U/mL), while synthesizing HMW levan (*M*_W_ = 2.3 × 10^6^ Da) at a low enzyme concentration (0.1 U/mL) [15]. Additionally, sucrose concentration could also affect product specificity of LS. Relatively high initial sucrose concentrations usually result in the synthesis of FOSs or LMW levan, while lower initial sucrose concentrations favor HMW levan production [16]. On the contrary, the *E*. *amylovora* LS [5] generated FOSs (DP 2–6) at a low sucrose concentration (200 mM), while it synthesized HMW levan at a high sucrose concentration (>500 mM). In addition to enzyme and substrate concentrations, temperature could also affect the product specificity. Lowering temperature was found to favor the T reaction of LS. For instance, the production of HMW levan from *Z*. *mobilis* LS was increased when the temperature was decreased from 40 to 4 °C [17].

In this work, a novel LS from the mesophilic bacteria *Pseudomonas orientalis* (Psor-LS) was screened on a *C*. *diazotrophica* LS (Cedi-LS) template. As a result, the Psor-LS showed maximum activity at 65 °C, much higher than the other LSs. The Psor-LS retained 46% of its initial activity at 55 °C for 9 h and 50% at 45 °C for 60 h, exhibiting excellent thermostability. Notably, two thermostable LSs showed a great difference in their product specificity. The Cedi-LS could produce FOSs, LMW (*M*_W_ = 4.1 × 10^4^ Da), and HMW (*M*_W_ = 1.8 × 10^6^ Da) levan in the reaction mixture, while the Psor-LS would specifically produce FOS and HMW (*M*_W_ = 1.4 × 10^6^ Da) levan rather than LMW levan. In particular, temperature was proposed to be significant to the product distribution of Cedi-LS and Psor-LS. When the temperature was changed from 65 to 35 °C, Cedi-LS tended to produce HMW with an increased *M*_W_ of 8.4 × 10^6^ Da. By contrast, at 35 °C, Psor-LS would produce more FOSs and significantly decrease the HMW levan. This study examines the effect of temperature on the LS product specificity and proposes a thermostable LS suitable for the HMW levan polymer and levan-type FOSs production.

## 2. Materials and Methods

### 2.1. Chemicals, Reagents, and Strains

*Escherichia coli* DH5α and BL21 (DE3) cells, Isopropyl β-D-1-thiogalactopyranoside (IPTG), ampicillin sodium, Luria–Bertani (LB) medium, and other chemicals of analytical grade were purchased from Sangon Biotech Co., Ltd. (Shanghai, China). Standards, including sucrose, glucose, and fructose, were purchased from Sigma (St. Louis, MO, USA) for high-performance liquid chromatography (HPLC) analysis.

### 2.2. Expression and Purification of Psor-LS

The genomic DNA of *P*. *orientalis* is available on the NCBI database with the GenBank accession number ASM385204v1, which revealed a putative gene encoding levansucrase. The gene was fused with the pET-22(+) vector using two restriction sites *Nde* I and *Xho* I, at the 5′- and 3′- terminus, which were commercially synthesized by Generay Biotech Co., Ltd. (Shanghai, China). A 6 × histidine-tag was designed at the 3′-terminus for purification via Ni^2+^ affinity chromatography.

For levansucrase expression, the recombinant plasmid was transformed into competent *E. coli* BL21 (DE3) cells, which were then inoculated into 200 mL of LB broth containing ampicillin (100 μg/mL broth) for the selection of transformants. The cells were cultivated at 37 °C with shaking at 200 rpm until the optical density at 600 nm (OD_600_) reached 0.6–0.8. Then, isopropyl β-D-1-thiogalactopyranoside (IPTG) was added into the broth with the final concentration of 1 mM, and the target enzyme expression was performed at 28 °C with shaking at 200 rpm for 6~7 h.

The bacteria were collected by centrifugation at 6000× *g* for 5 min, then disrupted in 15 mL lysis buffer (50 mM sodium phosphate buffer with 100 mM NaCl, pH 7). After being disrupted by ultrasonication, the supernatant was centrifuged at 8000× *g* and 4 °C for 10 min and filtered through a 0.45 μm Millipore filter. The enzyme was purified via Ni^2+^-affinity chromatography using Ni-IDA-Sefinose resin (Sangon Biotech Co. Ltd., Shanghai, China). A binding buffer (50 mM sodium phosphate buffer with 100 mM NaCl, pH 7.0), washing buffer (50 mM sodium phosphate buffer with 100 mM NaCl and 50 mM imidazole, pH 7.0), and elution buffer (50 mM sodium phosphate buffer with 100 mM NaCl and 500 mM imidazole, pH 7.0) were used to equilibrate the chromatography column, wash out non-specific proteins and elute target proteins, respectively. Then, the collected enzyme solution was dialyzed against 50 mM sodium phosphate buffer (pH 7.0) for more than 18 h to remove imidazole. SDS-PAGE examined the subunit molecular mass of Psor-LS with 12% (*w*/*v*) separation gel and 5% stacking gel, and the protein bands were stained with Coomassie Brilliant Blue R250. The Bradford assay [18] estimated the protein concentration using bovine serum albumin as the standard.

### 2.3. Enzyme Activity and the Ratio of Transfructosylation Activity to Hydrolytic Activity (T/H) Assay

The reaction systems of Psor-LS were set as 1 mL containing 30% (*w*/*v*) sucrose and 10 μg enzyme. The activity of Psor-LS was determined at 65 °C for 15 min. The reaction was stopped in boiling water for 10 min. One unit of total and hydrolytic activity was designated as the amount of enzyme catalyzing the release of 1 μmol glucose and fructose per minute, respectively. The transfructosylation activity was the subtraction between total and hydrolysis activity.

The ratio of transfructosylation activity to hydrolytic activity (T/H) of Psor-LS was determined at different concentrations of substrate (from 10% to 60%), different pH (4.0–9.0), and different temperatures (30–80 °C). The glucose and fructose contents of the reaction mixture were analyzed by high-performance liquid chromatography (HPLC). The Waters e2695 system (Waters Corporation, MA, USA) has a Waters 2414 RI detector and a Sugar-Pak I column (6.5 mm × 300 mm, Waters, USA). The column temperature and the mobile phase velocity were set as 85 °C and 0.4 mL/min.

### 2.4. Biochemical Characterization

Three types of buffers with different pH ranges were used to determine the influence of pH on the activity of Psor-LS: acetate buffer (pH 4.0–6.0, 50mM), sodium phosphate buffer (pH 6.0–7.5, 50 mM), and Tris-HCl (pH 7.5–9.0, 50 mM). The interval of each pH was set as 0.5. The reaction conditions were the same as those for the enzymatic activity assay.

The reactions were performed at various temperatures within 35–80 °C at intervals of 5 °C to measure the optimal temperature of Psor-LS. The other reaction conditions were the same as the enzymatic activity determination except for the temperature. The enzyme was incubated at 45, 55, and 65 °C for different time intervals to determine the thermostability of Psor-LS. The initial activity without pre-incubation treatment was taken as 100%. The formula calculated the half-life (*t*_1/2_) value, t_1/2_ = ln2/*k*_d_, and the inactivation rate constant (*k*_d_) was determined by the linear regression with the equation: ln (A_t_/A_0_) = *k*_d_ × t (A_t_: residual activity; A_0_: initial activity; t: incubation time).

Nano DSC III (TA Instrument, New Castle, DE, USA) was introduced to detect the denaturation temperature of this recombinant Psor-LS. The Nano-DSC was equipped with flow-through capillary cells loaded with a pipette mounted on both the inlet and outlet. The sodium phosphate (pH 7.0) buffer was used as the dialysis fluid overnight for the sample, and it was injected into the reference cell for buffer baseline determination. All samples were placed horizontally in a rack on an orbital shaker in a temperature-controlled incubator with gentle agitation. All samples must be degassed under a vacuum and loaded into the DSC. Scans were conducted by elevating temperatures from 20 °C to 100 °C, with a scan rate of 1 °C min^−1^. To measure molar heat capacities (Cp), sample scans were obtained from the subtraction of the buffer scans to measure molar heat capacities (Cp), and the experimental thermograms with protein concentration and the volume of the calorimeter cell were also normalized. The apparent denaturation temperature Tm values of Psor-LS were determined, and experiment data from Nano DSC III were analyzed using the Nano Analyze software package.

The kinetic parameters for Psor-LS against sucrose were determined at 65 °C. The reactions were prepared in potassium phosphate buffer (pH 6.5) containing sucrose 10-900 mM. The apparent Michaelis−Menten constant (Km) and the turnover number (kcat) were obtained through the nonlinear least square regression method (nonlinear regression Michaelis and Menten (http://biomodel.uah.es/en/metab/enzimas/MM-regresion.htm).

### 2.5. Optimization of Levan Production

A reaction containing 10–60% sucrose (*w*/*v*) was carried out to determine the optimal sucrose concentration. Under optimized conditions, different enzyme dosages, ranging from 5 to 100 μg/mL, were added to the reaction mixture to investigate the optimal enzyme dosages. The highest enzyme activity determined the optimal sucrose concentration. Differently, the optimal enzyme dosage was decided upon the benefit ratio, which is the ratio of the product formed in 15 min (including FOS and levan) to enzyme dosage. The biological production of Psor-LS was studied by measuring different saccharides at specific time intervals (0.5, 1, 2, 3, and 6 h).

### 2.6. Purification of Polysaccharide and FOSs

#### 2.6.1. Purification of Polysaccharides

Sevag reagent (N-butanol: chloroform = 1:4, *v*/*v*) was used to remove protein from the reaction mixture to purify fructans. The polysaccharides in the system were separated by ethanol (final concentration 60%, *v*/*v*) precipitation. Subsequent alcohol precipitation was used to increase the purity of polysaccharides (until HPLC could detect no monosaccharide and sucrose). The samples obtained were then dried via lyophilization using a LABCONCO FreeZone (LABCONCO Co., Kansas City, MO, USA).

#### 2.6.2. Purification of FOS

Ethanol present in the supernatant phases of alcohol precipitation in the Section 2.6.1 was removed by rotary evaporation. The FOS mixture was then purified by active carbon adsorption chromatography to remove glucose, fructose, and sucrose [19]. The chromatographic column was filled with treated activated carbon, and the column volume was 200mL. After loading the sample of 20 mL (flow rate of 1 mL/min), we let it stand for 1 h and balanced the column with purified water. The amount used was 2 column volume. Elution with 5%, 10%, 15%, 20%, and 30% concentrations and pure ethanol at a 1 mL/min flow rate was carried out. We collected one tube every 10 mL and performed determination of FOS content. The purified FOSs with high purity were then freeze-dried for 48 h.

### 2.7. Nuclear Magnetic Resonance (NMR) Analysis

The linkage between the fructosyl moieties of the biosynthesized fructans was characterized by ^1^H NMR and ^1^C NMR. About 35 mg of purified sample was dissolved in 500 μL deuterium oxide (D_2_O) by bathing at 60 °C. The ^1^H NMR and ^1^C NMR spectra were recorded using an AVANCE III 400 MHz NMR spectrometer (Brucker Co., Billerica, MA, USA) at 60 °C, which used acetone (^1^H = 2.225 ppm) and 1, 4-dioxan (^13^ C = 66.50 ppm) as internal reference standards.

### 2.8. M_W_ and Distribution Analysis

The *M*_W_ and distribution of levan were detected by high-performance gel filtration chromatography (HPGFC). The system was supplemented with a refractive-index detector and an Ultrahydrogel^TM^ Linear column (7.8 mm × 300 mm). The mobile phase is 0.1 N NaNO_3_ with a 0.5 mL/min flow rate. The *M*_W_ reference standards are Dextran T-2000 (*M*_W_ = 2 × 10^6^ Da, retention time is 14 min), Dextran T-300 (*M*_W_ = 3.0 × 10^5^ Da, retention time is 15.8 min), Dextran T-150 (*M*_W_ = 1.4 × 10^5^ Da, retention time is 16.4 min), Dextran T-10 (*M*_W_ = 9.7 × 10^3^ Da, retention time is 19 min) and Dextran T-5 (*M*_W_ = 2.7 × 10^3^ Da, retention time is 21 min). The detected temperature was 40 °C.

The degree of polymerization (DP) of FOS was determined by high-performance anion-exchange chromatography with pulsed amperometric detection (HPAEC-PAD). The column used in this system was a CarboPac PA200 column (3 mm × 250 mm) with the guard column CarboPac PA200 (3 mm × 50 mm). The eluent was 100 mM NaOH and 40 mM NaAc at the first 40 min, 100 mM NaOH and 400 mM NaAc at 40.1 min, and 100 mM NaOH and 40 mM NaAc between 40.1 and 60 min. The flow rate was 0.5 mL/min at 30 °C, and pH-Ag/AgCl as a reference electrode.

### 2.9. Molecular Dynamics Simulation

High-temperature molecular dynamics were performed using GROMACS (Version 2020.6) with an AMBER ff14SB force field [20,21]. The enzymes were solvated in TIP3P water and were relaxed through energy minimization to eliminate the error water insert. The system was heated to a pressure and temperature of 1 bar and 500 K using NVT and NPT ensemble balance with position constraint. The LINCS algorithm was used to constrain the hydrogen bonds in the system. The heavy atom of the protein is subjected to a position inhibition force with a constant of 1000 kJ mol^−1^ nm^−2^. After equilibrium, the final output of the NPT simulation was subjected to without-position limitation. The root-mean-square deviation (RMSD) of backbone atom positions per 5 ps were calculated and analyzed using GROMACS analysis tools.

The root-mean-square fluctuation (RMSF) was determined at 280 K, and the other conditions were the same as above. The final structure was simulated by molecular dynamics of 100 ns.

All assays were performed in triplicate. Data management and analysis were performed using GraphPad Prism 5.0 (GraphPad Software, San Diego, CA, USA). All data are presented as the mean ± standard error of the mean.

## 3. Results and Discussion

### 3.1. Computer-Aided Enzyme Screening

**Figure 1:**
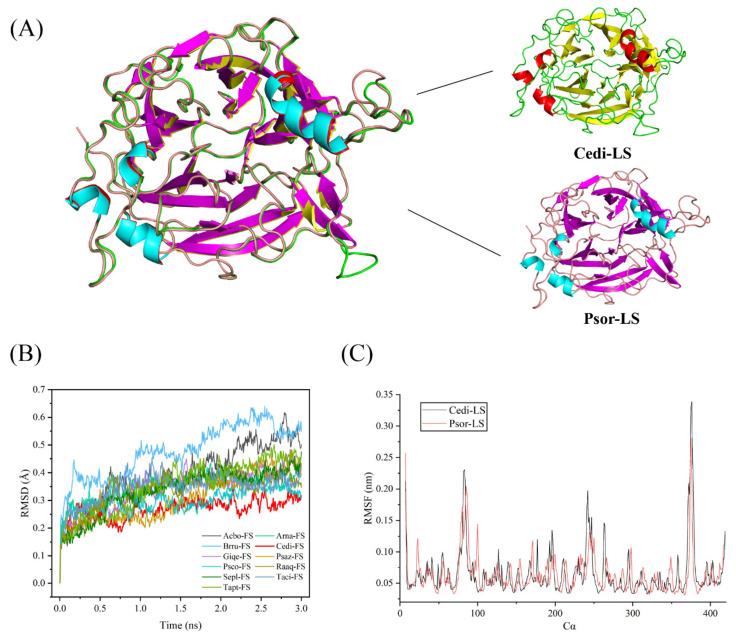
The structure and molecular dynamic simulation of Cedi-LS and Psor-LS. (**A**) The structure comparison of Cedi-LS and Psor-LS. The structures of Cedi-LS and Psor-LS are shown as carton. The superposition structure of Cedi-LS and Psor-LS are made by Pymol. (**B**) RMSD simulations of different microbial FSs at 500 K. (**C**) RMSF of the backbone Cα of modeled Cedi-LS and Psor-LS from the molecular dynamic simulation at 280 K.

Unlike the traditional BLAST tool, a computer-aided enzyme screening method combined with the Enzyme miner online server (https://loschmidt.chemi.muni.cz/enzymeminer/custom-sequences) [22] was employed to screen out the potential thermostable LS. High-temperature molecular dynamics simulations predicted the flexibilities of enzyme orthologs and thermostability. The RMSD value of the different microbial FSs is shown in Figure 1B, which can quantify the backbone atom movements of the protein. The Genbank accession numbers of these FSs are listed in Table S1. As a result, the LS from *C. diazotrophica* (Cedi-LS) exhibited the lowest RMSD value among all the enzymes, which means that Cedi-LS with the most rigid structure might have the best thermostability. Subsequently, a novel LS from mesophilic bacteria *P. orientalis* (Psor-LS) was screened on the template of Cedi-LS.

The 3D structures of LSs were homologically modeled employing the crystal structures of *E*. *tasmaniensis* LS (PDB: 6FRW) as their template in the SWISSMODEL online server (https://swissmodel.expasy.org/) [23]. As shown in Figure 1A, Psor-LS showed a high coincidence degree in structure with Cedi-LS, except in loops 1 and 8. The RMSD value between two of the LSs was 0.117. The RMSF was used to study the movement of each residue in the enzyme and determine the flexibility of a particular region in the protein. As shown in Figure 1C, the RMSF results of Psor-LS and Cedi-LS showed a similar pattern, and the maximum D-value did not exceed 0.06 nm. Meanwhile, Cedi-LS and Psor-LS both showed low RMSF values in the whole structure (below 0.35 nm), indicating a very close relationship between the crystal structures of Cedi-LS and Psor-LS.

### 3.2. Expression and Purification of Psor-LS

**Figure 2:**
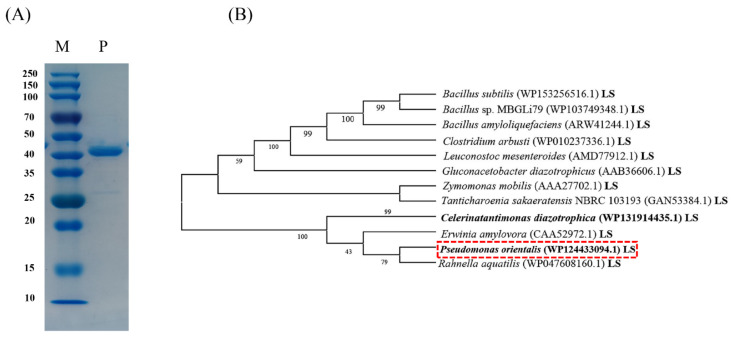
SDS-PAGE and phylogenetic tree. (**A**) Lane P represents the purified LS from *P*. *orientalis*, and lane M represents the protein marker with standard enzymes with the following molecular weights: 250, 150, 100, 70, 50, 40, 35, 25, 20, 15, and 10 kDa. (**B**) Phylogenetic tree of LSs from different sources. The phylogenetic tree was constructed by MEGA 5.1. The content in the bracket is the accession numbers in the GenBank database.

The sequence of the gene encoding the Psor-LS has been deposited in the GenBank database. SDS-PAGE analysis of the recombinant protein from *P*. *orientalis* indicated a band around 44 kDa (Figure 2A), consistent with the calculated molecular weights of the LS (ExPASy Computer *M*_w_ tool, https://web.expasy.org/compute_pi/), suggesting a triumphant expression of the target enzyme in *E*. *coli*.

Amino acid sequence identity analysis was performed by EMBI 225 (https://www.ebi.ac.uk/Tools/services/web/tool). The Psor-LS showed the highest identity of 80% with the *R. aquatilis* LS and had more than 70% identity to LSs from *C. diazotrophica*, *Brenneria* sp. EniD312 and *E. amylovora*. By contrast, the Psor-LS showed the lowest identity of 24% with the *Leuconostoc mesenteroides* LS. They only had less than 30% identity to the LSs from *B*. *subtilis*, *B. amyloliquefaciens*, and *Clostridium arbusti*. The evolutional relationship to LSs from different sources is shown in Figure 2B.

### 3.3. Effect of pH on the Activity and T/H Ratio of Psor-LS

**Figure 3:**
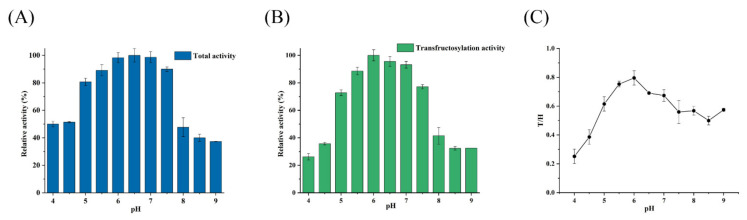
Effect of pH on the activity and T/H of the recombinant LS. (**A**) Effect of pH on the total activity of Psor-LS. (**B**) Effect of pH on the transfructosylation activity of Psor-LS. (**C**) Effect of pH on the T/H of Psor-LS. All of the values were the mean of triplicate experiments.

As shown in Figure 3A, Psor-LS showed relatively high activity (>80%) at pH values ranging from 5.0 to 7.5 but dropped when pH was below 5.0 or above 7.5. Figure 3B shows the effect of pH on the transfructosylation activity of Psor-LS. Unlike Cedi-LS, the Psor-LS was sensitive to pH since it exhibited the maximum transfructosylation activity at pH 6.0. However, less than 50% of the activity remained when pH was shifted from 6.0 to 4.5 or 8.0. Unlike Cedi-LS, the T/H of Psor-LS was lower than 1.0 in the whole pH range of 4.0 to 9.0, which means that the hydrolysis reaction was dominant for Psor-LS (Figure 3C). Most LSs exhibited optimal activity at slightly acidic (5.5) or neutral pH (7.0). For instance, the LS from *Brenneria goodwinii* [24] showed optimal activity at pH 5.5 and 6.0. The *L*. *mesenteroides* MTCC10508 LS had the highest activity at pH 5.5 [25].

### 3.4. Effect of Temperature on the Activity and T/H of Psor-LS

**Figure 4:**
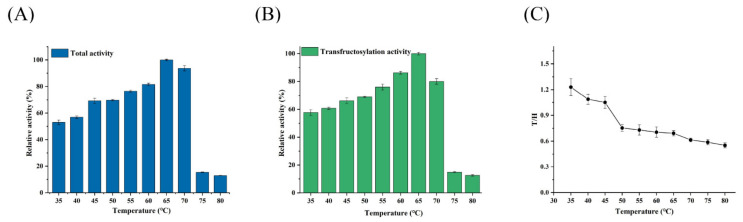
Effect of temperature on the activity and T/H of the recombinant LS. (**A**) Effect of temperature on the total activity of Psor-LS. (**B**) Effect of temperature on the transfructosylation activity of Psor-LS. (**C**) Effect of temperature on the T/H of Psor-LS. All of the values were the mean of triplicate experiments.

The effect of temperature on Psor-LS activity was measured at an optimal pH of 6.5. As a result, the optimal temperature of Psor-LS was 65 °C (Figure 4A), the same as Cedi-LS. Psor-LS could continue relatively high activity (>70%) at temperatures ranging from 45 to 70 °C, but this dropped when the temperature was above 75 °C. A slight decrease was observed below 45 °C, but Psor-LS could retain more than 50% of its relative activity at 35 °C. The variation in transfructosylation activity is shown in Figure 4B. Psor-LS could retain 80% of its transfructosylation activity at 60–70 °C, suggesting that Psor-LS exhibits excellent transfructosylation ability at high temperatures. Unlike Cedi-LS, a relatively high T/H value of Psor-LS (>1.0) was obtained below 45 °C (Figure 4C).

Many previous studies have shown that LS showed higher transfructosylation activities at lower temperatures, while hydrolysis activity shows the opposite. For example, the LS from *Z. mobilis* exhibited the highest transfructosylation activity at 30 °C, while its hydrolase activity optimal temperature was 50 °C [26]. The *L*. *reuteri* LTH5448 LS showed the highest transfructosylation and hydrolase activity at 35 °C and 45 °C, respectively [9]. This characteristic is also related to the T/H of LS. At higher temperatures (>45 °C), hydrolysis is the dominant reaction for *B. goodwinii* LS [24]. The LS from *L. reuteri* LTH5448 exhibited a higher hydrolysis ability above 50 °C, with its T/H below 1.0 [9]. By contrast, Psor-LS exhibited high transfructosylation activity at high temperatures (>55 °C), which was better than most LSs.

### 3.5. Thermostability Determination of Psor-LS

**Figure 5:**
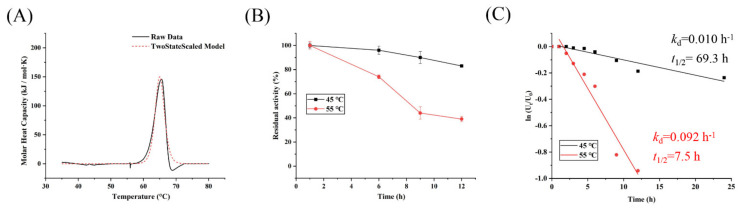
*T*_m_ and thermostability of recombinant LS. (**A**) *T*_m_ of Psor-LS. (**B**) Thermostability of Psor-LS at 45 and 55 °C. (**C**) The *t*_1/2_ value of Psor-LS at 45 and 55 °C.

Melting temperature (*T*_m_) is related to the structural stability of enzymes, and high *T*_m_ generally represents high structural stability and thermostability [27]. The *T*_m_ of Psor-LS was determined to be 65.1 °C (Figure 5A), which was significantly higher than that of FSs from *L*. *reuteri* 121 (50 °C) and IS from *L*. *gasseri* (55 °C) [27,28]. To further test the stability of Psor-LS at different temperatures, the enzyme was incubated at 45, 55, and 65 °C, respectively. Psor-LS retained 46% of initial activity at 55 °C for 9 h and half of the initial activity at 45 °C for 60 h (Figure 5B). The half-life was 69 h at 45 °C and 7.5 h at 55 °C (Figure 5C). However, Psor-LS was almost inactive when solely incubated at 65 °C for 10 min.

Except for the LS from *L. reuteri* LTH5448, most LSs showed low thermostability at temperatures above 50 °C (Table 1). For instance, the *B*. *licheniformis* RN-01 LS retained less than 50% of its initial activity after 1 h of incubation at 50 °C [29]. The LS from *Bacillus* sp. TH4-2 lost 50% of its initial activity at 60 °C for 30 min [30]. The LS from *B*. *subtilis* NRC could retain 60% of its activity after incubating at 50 °C for 2 h [31]. Before this study, the LS from *Geobacillus stearothermophilus* was reported as the most thermostable LS since it could retain more than 95% of initial activity at 4-47 °C for 6 h [32]. However, the enzyme would rapidly lose activity at higher temperatures (57 °C).

### 3.6. Kinetic Parameters Determination

Differently from previous studies, the kinetics of fructose and glucose “release” in the reaction have been calculated. The rate of glucose release is the hydrolysis rate plus the transferase reaction rate. The *K*_m_ of hydrolysis and transferase reaction is expressed as “*K*_m_^H^” and “*K*_m_^T^”, respectively [36]. The kinetic parameters of Cedi-LS and Psor-LS are shown in Table 2. The determined *K*_m_^H^ and *K*_m_^T^ values of Cedi-LS were 57 ± 2 and 202 ± 7 mM, respectively. Cedi-LS showed higher *k*_cat_ values for transfructosylation than for hydrolysis, and its *k*_cat_^H^/*k*_cat_^T^ was 1.35, significantly higher than that of *B. subtilis* LS (0.37) [36]. The *K*_m_^H^ (117 ± 8 mM) and *k*_cat_^H^ (620 ± 12 s^−1^) of Psor-LS were higher than Cedi-LS, indicating that Psor-LS is more favorable to hydrolyze sucrose than Cedi-LS. Nevertheless, the kinetic parameter of the transferase reaction of Psor-LS did not conform to the nonlinear least square regression method.

### 3.7. The Effect of Sucrose Concentration on the Activity and T/H of Psor-LS

**Figure 6:**
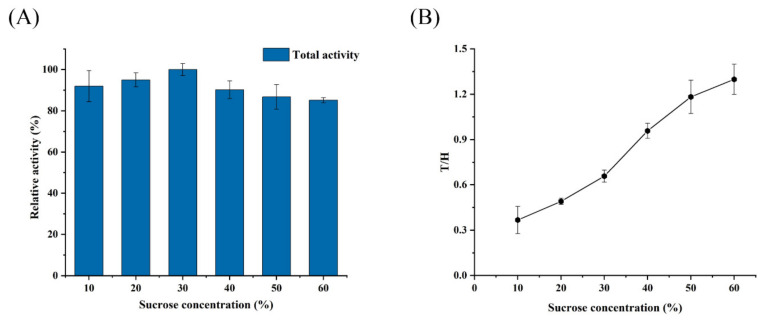
Effect of sucrose concentration on the activity and T/H of the recombinant LSs. (**A**) Effect of sucrose concentration on the total activity of Psor-LS. (**B**) Effect of sucrose concentration on the T/H of Psor-LS. All of the values were the mean of triplicate experiments.

Sucrose concentration is an essential factor in the activity and T/H of LSs. As a result, Psor-LS exhibited the highest activity at 30% sucrose (Figure 6A). The Psor-LS could retain over 80% of its activity at substrate concentrations ranging from 10 to 60%, suggesting that Psor-LS showed a broad sucrose concentration spectrum for its activity. In this study, the total activity of Psor-LS could be saturated at 30% sucrose. A similar result was reported in the LSs from *B*. *methylotrophicus* SK 21.002 [7] and *Z. mobilis* [37]. The “E-F⋅F-G” complex accumulation and the nonproductive binding are possibilities for this inhibitory phenomenon [37]. Although the activity was decreased, the T/H of the Psor-LS showed an upward trend with the increase in sucrose concentration (Figure 6B). The Psor-LS showed relatively high T/H (>1) at 40% sucrose and exhibited the highest T/H at 60% sucrose (1.3). By comparison, the *E*. *amylovora* LS had a T/H of about 4 at 100 mM sucrose, and its transfructosylation reaction could reach a maximum of 97% at 1.5 M sucrose [5].

### 3.8. Effect of Enzyme Concentration on the Levan Production and T/H of Psor-LS

**Figure 7:**
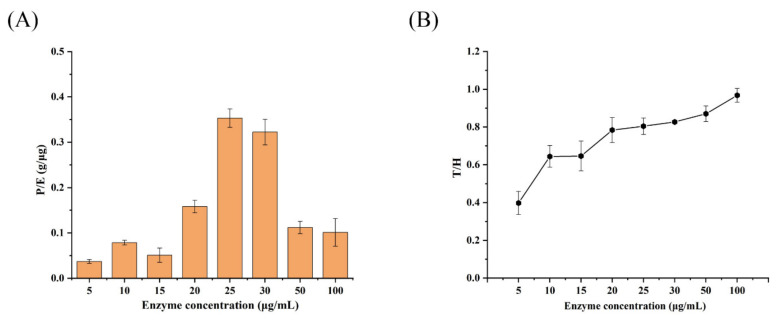
Effect of enzyme concentration on the P/E and T/H of the recombinant LS; P/E is the ratio of levan production and enzyme concentration. (**A**) Effect of enzyme concentration on P/E of Psor-LS. (**B**) Effect of enzyme concentration on T/H of Psor-LS. All of the values are the mean of triplicate experiments.

Different enzyme dosages ranging from 10 to 100 μg/mL at 30% sucrose were employed to optimize the levan production. Since enzyme concentration was related to levan production, the ratio of levan production and enzyme concentration (P/E) was evaluated as “input–output” in this study. As a result, the highest P/E value of Psor-LS was exhibited at 25 μg/mL enzyme, suggesting that both enzyme and substrate were the maximum output in this enzyme dosage (input) (Figure 7A). A comparable P/E ratio was observed at 30 μg/mL enzyme concentration compared to 25 μg/mL. However, it dropped remarkably when enzyme concentration was below 25 or above 30 μg/mL. The Psor-LS showed a growing T/H with increased enzyme concentration (Figure 7B), and it exhibited an equivalent transfructosylation and hydrolysis reaction at 100 μg/mL enzyme. A similar result for IS from *Lactobacillus jensenii* was reported when the T/H was increased with increased enzyme dosage [38]. By contrast, the *B*. *subtilis* LS showed high T/H (2.7) at 0.1 U/mL enzyme concentration but low T/H (0.7) when enzyme concentration is increased to 10 U/mL.

### 3.9. Biological Production of Psor-LS

**Figure 8:**
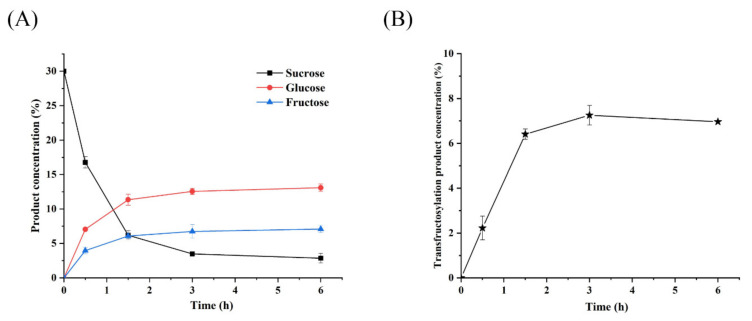
Biotransformation process and conversion ratio of recombinant LS. (**A**) The variation in sucrose, glucose, and fructose concentration in the biotransformation process of Psor-LS. (**B**) The variation in conversion ratio in the biotransformation process of Psor-LS. All of the values are the mean of triplicate experiments.

The biotransformation process of Psor-LS is shown in Figure 8A. Rapid sucrose consumption was shown in the first 1.5 h. The consumption rate slowed down in the next 1.5 h. After 3 h, the sucrose concentration was almost unchanged, consuming at a very low rate. When the reaction reaches equilibrium, the maximum conversion ratio of the transfructosylated product to sucrose was 29.2% at 3 h (Figure 8B). Like Cedi-LS, the product produced by Psor-LS decreased slowly after 3 h. By comparison, the LSs from *B*. *methylotrophicus* SK 21.002 and *B*. *licheniformis* NS032 could also produce levan effectively, and their conversion ratios were 33 and 25%, respectively [7,39].

As reported, the T/H of Cedi-LS was 1.3 at 65 °C, higher than that of Psor-LS (0.8). T/H is considered to reflect the transfructosylation ability of LS and continue to the product distribution of LS. For instance, the *B*. *subtilis* LS showed higher T/H (2.7) at 0.1 U/mL enzyme concentration and produced HMW levan. When the enzyme concentration was 1 U/mL, the enzyme produced LMW levan with lower T/H (1.0) [15]. As shown in Figure 8A, the residual fructose in the Psor-LS system is significantly higher than that in Cedi-LS, consistent with its relatively lower T/H value. Meanwhile, the residual sucrose and glucose in Psor-LS system are lower than that in Cedi-LS, which indicates that Psor-LS has higher glucose utilization than Cedi-LS.

### 3.10. Effect of Temperature on the Product Distribution of Cedi-LS and Psor-LS

**Figure 9:**
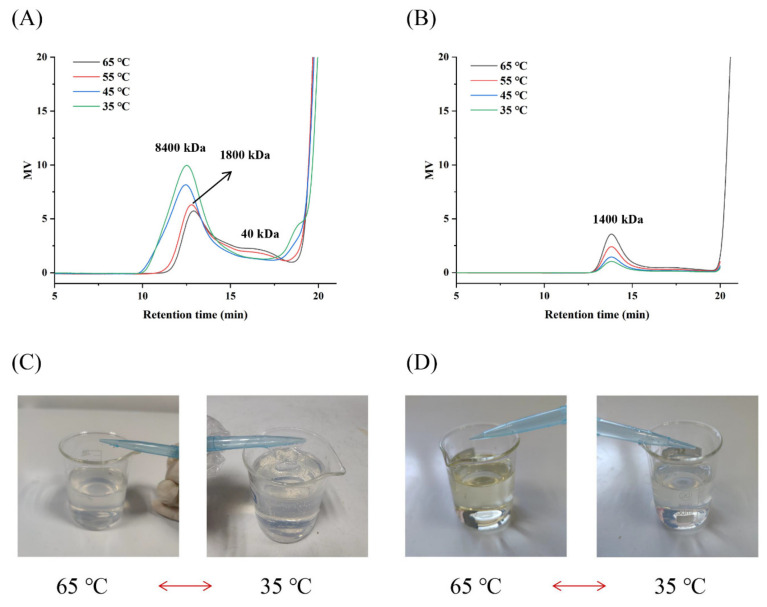
Effect of temperature on the product distribution and solution status of the recombinant LSs. (**A**) Effect of temperature on the product distribution of Cedi-LS. (**B**) Effect of temperature on the product distribution of Psor-LS. (**C**) Effect of temperature on the reaction mixture of Cedi-LS. (**D**) Effect of temperature on the reaction mixture of Psor-LS.

Many factors were considered to be potential reasons for the product specificity of LSs, such as sucrose concentration [5] and enzyme concentration [15]. The temperature could also affect the product specificity [17]. However, how temperature could affect the product specificity of LS remains unclear. To investigate the effect of temperature on the product distribution of LS, we reduced the reaction temperature from 65 to 35 °C. At 35 °C, the product conversion ratios of Cedi-LS and Psor-LS were 41.4 and 37.1%, respectively. The T/H values of the two enzymes were 2.3 and 1.0. Moreover, the viscosity of the reaction solution of Cedi-LS was increased when the temperature was decreased to 45 and 35 °C. At 2 h reaction time, the solution showed a “gel-similar” phenomenon (Figure 9C), which is much different from that of the Psor-LS solution (Figure 9D).

To further determine the possible change in product distribution at different temperatures, the reaction mixture components were analyzed by HPGFC in detail (Figure 9A,B). At its optimal temperature of 65 °C, the Cedi-LS could simultaneously produce FOS, LMW (4.1 × 10^4^ Da), and HMW (1.8 × 10^6^ Da) levan in the reaction mixture. On the contrary, the Psor-LS specifically produced FOS and HMW (1.4 × 10^6^ Da) levan without LMW levan. When the temperature was decreased, the levan produced by Cedi-LS showed a higher *M*_W_ that reached 8.4 × 10^6^ Da at 35 °C. Simultaneously, a low temperature results in higher production of HMW levan. Therefore, the increase in *M*_W_ and production of HMW levan were supposed to result in a higher viscosity of the reaction solution, as shown in Figure 9C. The low temperature increased the HMW levan in many LSs [40]. For instance, the LS from *R*. *aquatilis* ATCC 33071 mainly produced FOS at 55–60 °C, while it synthesized HMW levan (1 × 10^6^ Da) at low temperature (37 °C) [41]. The production of HMW levan increased obviously at low temperatures (4 °C) in *Z. mobilis* LS [17]. On the contrary, the production of HMW levan in Psor-LS decreased as the temperature decreased. This means that lower temperatures promoted the synthesis of FOS in Psor-LS. As far as the authors are concerned, this is the first LS that prefers to produce FOSs rather than HMW levan when the temperature is decreased. Moreover, the *M*_W_ of the levan from Psor-LS was not changed, indicating that the temperature has different effects on the product distribution of Cedi-LS and Psor-LS.

### 3.11. Product Purification and Analysis

**Figure 10:**
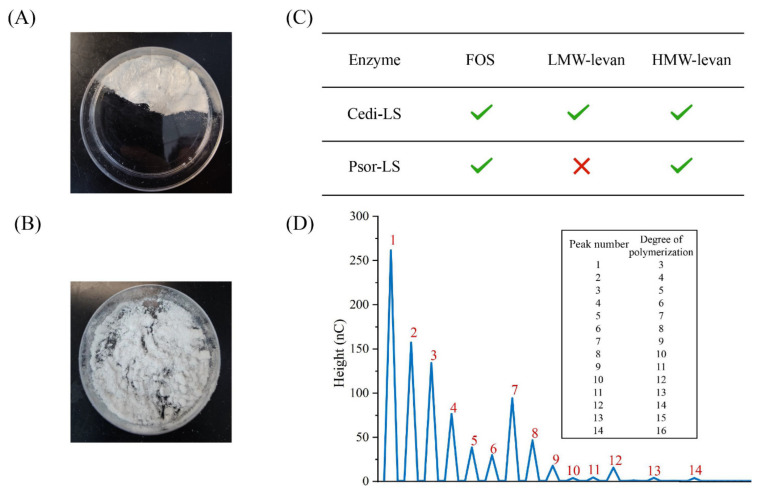
Products from the recombinant LSs. (**A**) The purified HMW levan from Psor-LS. (**B**) The purified FOS from Psor-LS. (**C**) The component analysis of products from Cedi-LS and Psor-LS. (**D**) The HPIC spectrogram of purified FOS from Psor-LS.

The residual enzyme in the mixture was removed by Sevage reagent, and the polysaccharide was separated by multiple ethanol precipitation. When the final ethanol concentration was 60%, the polysaccharide produced from Psor-LS was obtained. The obtained precipitate was vacuum freeze-dried for 48 h to remove moisture altogether (Figure 10A). The products of Psor-LS were identified as *β*-(2, 6) levan and levan-type FOSs by the NMR analysis (Figure S1). The ^1^H spectrograms were compared with those of *L. reuteri* LTH5448 LS and *L. jensenii* IS [9,38]. Meanwhile, the ^13^C chemical shifts reported for biosynthesized levan are compared in Table S2. The result revealed that the polysaccharide synthesized by Psor-LS was *β*-(2, 6) levan.

The *M*_W_ of levan synthesized by Psor-LS was 1.4 × 10^6^ Da (65 °C). Generally, the *M*_W_ of HMW levan produced by LS from different microorganisms were different, such as the LSs from *T*. *sakaeratensis* (1.0−6.8 × 10^5^ Da) [42], *A*. *diazotrophicus* SRT4 (2.0 × 10^6^ Da) [43] and *Bacillus aryabhattai* (5.3 × 10^7^ Da) [44]. FOS was purified by activated carbon chromatography to remove the sucrose and monosaccharides and dried in a freeze dryer for 48 h. The purified FOS is shown in Figure 10B. The purity of FOS produced by Psor-LS with DP ≤ 16 could reach more than 90%, but quantitative comparison cannot be carried out due to the significant loss in the purification process (Figure 10D).

## 4. Conclusions

In this study, a novel thermostable LS from *P*. *orientalis* was identified. The Psor-LS retained 46% of its initial activity at 55 °C for 9 h and 50% at 45 °C for 60 h. Meanwhile, there are noticeable differences in the product distribution between the Cedi-LS and Psor-LS. The *M*_W_ of levan synthesized by Cedi-LS was increased from 1.8 × 10^6^ Da (65 °C) to 8.4 × 10^6^ Da (35 °C). On the contrary, the decrease in temperature did not significantly affect the product distribution of Psor-LS. At 65 ℃, the Psor-LS would specifically produce FOSs and HMW levan without LMW levan. Notably, at 35 °C, the reaction equilibrium of Psor-LS from sucrose (30%) was 37%, and a certain amount of FOS (DP ≤ 16) was obtained among them.

## Figures and Tables

**Table 1:** Thermostability of LSs from different microorganisms.

Microorganisms	Optimal Temp. (°C)	Thermostability	Reference
*P*. *orientalis*	65	The half-life was 69 h at 45 °C and 7.5 h at 55 °C.	This study
*B*. *licheniformis* RN-01	50	Half of the initial activity was lost after 1 h at 50 °C.	[29]
*Bacillus* sp. TH4-2	60	Half of the initial activity was lost after 30 min at 60 °C.	[30]
*G*. *stearothermophilus*	57	More than 95% of the initial activity was retained at 4-47 °C for 6 h.	[32]
*Z*. *mobilis*	30	The activity lost at 50 °C for 15 min.	[33]
*Brenneria* sp. EniD312	45	The half-life was 2 h at 45 °C and 1.2 h at 55 °C	[34]
*B*. *goodwinii*	40	The activity lost after 0.5 h of incubation at 50 °C	[24]
*L*. *reuteri* LTH5448	35	The activity remained 63.8% at 55 °C for 12 h	[9]
*Bacillus subtilis* NRC	45	The activity remained 60% at 50 °C for 12 h	[31]
*B*. *circulans*	35	The half-life was 130 min at 50 ℃	[35]

**Table 2:** Kinetic parameters of Cedi-LS and Psor-LS.

LS	*K*_m_ (mM)		*k*_cat_ (s^−1^)		*k*_cat_/*K*_m_ (mM^−1^ s^−1^)	
	Hydrolysis	Transfer	Hydrolysis	Transfer	Hydrolysis	Transfer
Cedi-LS	57 ± 2	202 ± 7	332 ± 22	449 ± 13	5.80	2.23
Psor-LS	117 ± 8	-	620 ± 12	-	5.27	-

## Data Availability

Data presented in this study are available on request from the corresponding author.

## Supplementary Materials

The following supporting information can be downloaded at: https://www.mdpi.com/article/10.3390/polym15061435/s1, Figure S1: Nuclear magnetic resonance (NMR) analysis spectrogram of the products from Psor-LS; Table S1: The genbank number and sequnce length of different microbial FSs; Table S2: ^13^C chemical shifts reported for biosynthesized levan [7,45].
