# Supplementary material for: Identification of a Thermostable Levansucrase from Pseudomonas orientalis That Allows Unique Product Specificity at Different Temperatures
Source: Polymers (Basel). 2023 Mar 14;15(6):1435. doi: 10.3390/polym15061435 (PMC10058814; doi:10.3390/polym15061435)
Supplement: Supplementary file 1 [file polymers-15-01435-s001.zip › polymers-2199077-supplementary.pdf]

Supplementary Materials

Table S1. The genbank number and sequnce length of different microbial FSs.

Microbial source	Genbak number	Sequnce length
Acinetobacter boissieri	WP_092750189.1	427
Arsenophonus nasoniae	WP_034249982.1	415
Brenneria rubrifaciens	WP_137712490.1	436
Celerinatantimonas diazotrophica	WP_131914435.1	419
Gibbsiella quercinecans	WP_095849051.1	426
Pseudomonas azotoformans	WP_141606063.1	416
Pseudomonas coronafaciens	WP_147476483.1	402
Rahnella aquatilis	WP_047611456.1	415
Serratia plymuthica	WP_122289004.1	416
Tatumella citrea	WP_087487124.1	419
Tatumella ptyseos	WP_029990647.1	419

Table S2. ^{13}C chemical shifts reported for biosynthesized levan [7,45].

Microorganisms	C-1	C-2	C-3	C-4	C-5	C-6	References
B. licheniformis ANT 179	62.58	106.80	78.96	77.89	82.89	65.99	[45]
B. methylotrophicus SK 21.002	61.20	104.66	77.51	76.10	80.77	63.94	[7]
P. *orientalis*	61.13	104.68	77.33	75.32	80.46	63.61	This study

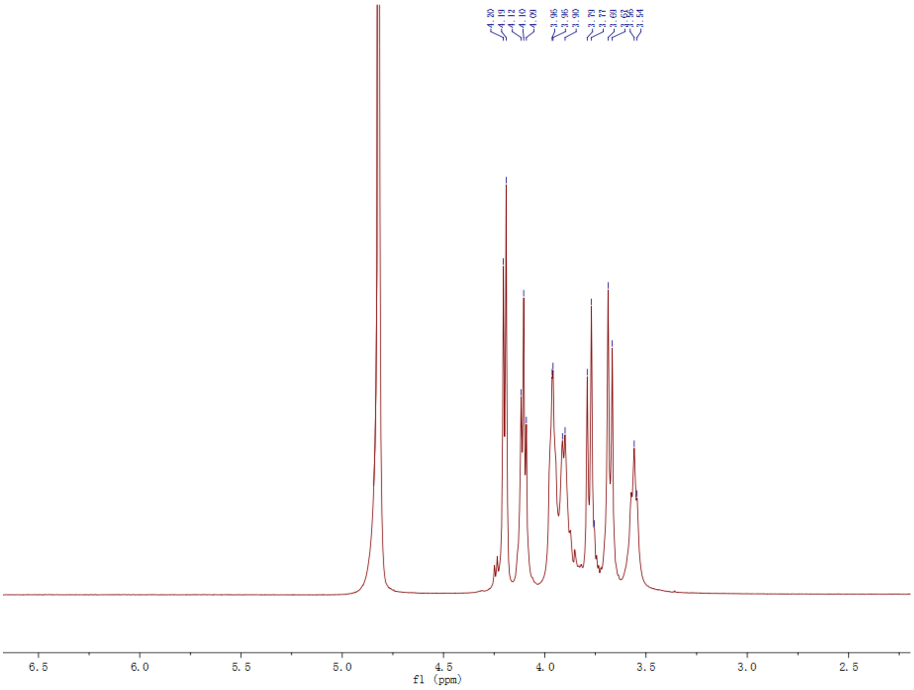

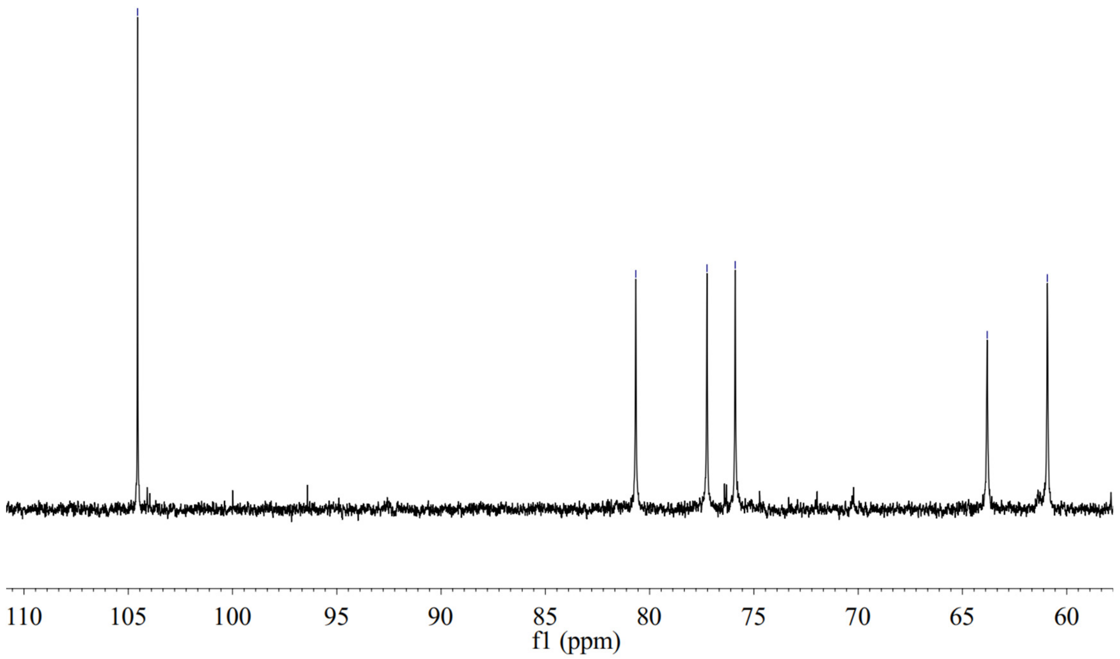

Figure S1. Nuclear magnetic resonance (NMR) analysis spectrogram of the products from Psor-LS.
